# An Integrated Approach of Belief Rule Base and Deep Learning to Predict Air Pollution

**DOI:** 10.3390/s20071956

**Published:** 2020-03-31

**Authors:** Sami Kabir, Raihan Ul Islam, Mohammad Shahadat Hossain, Karl Andersson

**Affiliations:** 1 Department of Computer Science, Electrical and Space Engineering, Luleå University of Technology, SE-931 87 Skellefteå, Sweden; raihan.ul.islam@ltu.se (R.U.I.); karl.andersson@ltu.se (K.A.); 2 Department of Computer Science & Engineering, University of Chittagong, Chattogram 4331, Bangladesh; hossain_ms@cu.ac.bd

**Keywords:** BRBES, Deep Learning, integration, sensor data, predict

## Abstract

Sensor data are gaining increasing global attention due to the advent of Internet of Things (IoT). Reasoning is applied on such sensor data in order to compute prediction. Generating a health warning that is based on prediction of atmospheric pollution, planning timely evacuation of people from vulnerable areas with respect to prediction of natural disasters, etc., are the use cases of sensor data stream where prediction is vital to protect people and assets. Thus, prediction accuracy is of paramount importance to take preventive steps and avert any untoward situation. Uncertainties of sensor data is a severe factor which hampers prediction accuracy. Belief Rule Based Expert System (BRBES), a knowledge-driven approach, is a widely employed prediction algorithm to deal with such uncertainties based on knowledge base and inference engine. In connection with handling uncertainties, it offers higher accuracy than other such knowledge-driven techniques, e.g., fuzzy logic and Bayesian probability theory. Contrarily, Deep Learning is a data-driven technique, which constitutes a part of Artificial Intelligence (AI). By applying analytics on huge amount of data, Deep Learning learns the hidden representation of data. Thus, Deep Learning can infer prediction by reasoning over available data, such as historical data and sensor data streams. Combined application of BRBES and Deep Learning can compute prediction with improved accuracy by addressing sensor data uncertainties while utilizing its discovered data pattern. Hence, this paper proposes a novel predictive model that is based on the integrated approach of BRBES and Deep Learning. The uniqueness of this model lies in the development of a mathematical model to combine Deep Learning with BRBES and capture the nonlinear dependencies among the relevant variables. We optimized BRBES further by applying parameter and structure optimization on it. Air pollution prediction has been taken as use case of our proposed combined approach. This model has been evaluated against two different datasets. One dataset contains synthetic images with a corresponding label of PM_2.5_ concentrations. The other one contains real images, PM_2.5_ concentrations, and numerical weather data of Shanghai, China. We also distinguished a hazy image between polluted air and fog through our proposed model. Our approach has outperformed only BRBES and only Deep Learning in terms of prediction accuracy.

## 1. Introduction

The Internet of Things (IoT) refers to a global network of objects around us, which can interact with each other through embedded systems. It provides infrastructure to capture, store, and process data coming from various sensors [1]. Radio Frequency IDentification (RFID) and Wireless Sensor Network (WSN) technologies are used to develop such embedded systems [2]. IoT is creating immense opportunities for novel applications of sensor data [3]. The sources of such sensor data are heterogeneous and their integration is also a complex task [4]. Predictive output is obtained by applying reasoning over these heterogeneous sensor data. There are numerous use cases of sensor data streams where prediction facilitates precautionary measures to protect both humans and properties. For example, predicting air quality by reasoning over the sensor data of major air pollutants, e.g., PM_2.5_, PM_10_, CO, O_3_, etc. [5]. The concentration of these air pollutants can be taken from ground-level sensors as well as satellite-based maps. In addition to the concentration level, satellite maps provide spatial distribution of the concerned pollutant over a certain geographical area [6]. Moreover, spatial concentration field of air pollutants can be created by deterministic air quality modelling. This modelling applies various data assimilation techniques to generate such spatial distribution [7]. Outdoor air pollution causes around three-million deaths every year [8]. Presently, air pollution ranks fourth globally to trigger human health casualties [9]. It inflicts a loss of around US$ 5 trillion annually on world economy [10]. Accordingly, computing accurate prediction of air pollution can improve people’s living standard significantly. Being motivated by this, we present air pollution prediction as use the case of our proposed integrated approach. We have considered the sensor data of PM_2.5_ concentrations in order to predict air pollution level with reference to Air Quality Index (AQI). Here, PM_2.5_ refers to the Particulate Matter with diameter less than 2.5 micrometers. AQI is a numerical scale with a corresponding color code and it is divided into several specific ranges [11]. In addition to the pollution level, this index warns citizens of potential health risk, which is critical for children, elderly people, and people with respiratory diseases. We use the breakpoint table, which was developed by the U.S. Environmental Protection Agency (EPA) based on six common air pollutants, to calculate AQI against the level of PM_2.5_ [12]. 

Prediction can be computed in two ways. One is knowledge-driven approach and the other is a data-driven approach [13]. Knowledge-driven approach is formulated by an expert system. This expert system comprises of two parts: knowledge base and inference engine. The knowledge base is constructed by if-then rules, rather than the conventional procedural code, in order to demonstrate the rules and facts. Inference engine makes reasoning over input data against these rules to deduce predictive output. Belief Rule Based Expert System (BRBES), fuzzy logic, MYCIN [14], PERFEX [15] are some of the knowledge-driven approaches. However, predictive output becomes unreliable due to deceptive or erroneous nature of input sensor data. Low battery power, computational and memory constraints, inadequate communication bandwidth, malicious attacks, and harsh environments are the factors that cause missing, duplicate, or inconsistent sensor data, which results in uncertainties [16,17]. Similarly, images that are captured by a camera sensor can become blurred due to inclement weather. Snow-covered or water-marked glasses of camera will result in hazy images. Hence, addressing such uncertainties of sensor data is of paramount importance in improving prediction accuracy. In terms of handling different types of uncertainties, especially ignorance, BRBES outperforms other knowledge-driven approaches [18]. Knowledge base is developed by Propositional Logic (PL) and First Order Predicate Calculus (FOPC). Forward Chaining (FC) and Backward Chaining (BC) are deployed to construct the inference engine. Nonetheless, PL and FOPC constitute assertive knowledge. Therefore, they cannot capture uncertainties of knowledge [18]. BRBES deploys Evidential Reasoning (ER) as its inference engine to get over this limitation [19].

The data-driven approach learns representation of external data independently. It infers predictive output by discovering hidden representation of data, such as, sensor data, historical data. There is no rule base in data-driven approach. Rather, it learns by examples to produce actionable insight. Machine Learning, which falls under AI, is a data-driven approach. Machine Learning builds a mathematical/statistical model of training data to make predictions without being explicitly programmed to perform the task. Image recognition, object detection, and speech recognition are some of its application areas, where there is no particular rule base to achieve predictive output. There are three major types of Machine Learning algorithms—Supervised learning, Unsupervised learning, and Reinforcement learning. Supervised learning algorithms build a statistical model of labeled data, which consists of both inputs and desired outputs. Testing data are used to calculate validation accuracy of the model. Finally, the trained model predicts pattern with respect to new input data. Support Vector Machine (SVM), Classification and Regression Trees (CART), Naive Bayes (NB), and K-Nearest Neighbours (KNN) are some of the supervised learning algorithms [20]. Unsupervised learning algorithms learn hidden structure of unlabeled data. It predicts based on commonalities in new input. Apriori, K-means, and Principal Component Analysis (PCA) [21] are some of the unsupervised learning algorithms. Reinforcement learning uses trial and error process to decide next course of action with maximum reward. For example, a robot learns the collision-free path upon sensing the presence of obstacles [22].

However, Machine Learning cannot directly deal with natural raw data [23]. It does not possess feature extractor to turn raw data into proper internal representation or feature vector. On the other hand, Deep learning, which adopts neural network architecture, overcomes this shortcoming, as it can handle raw data on its own. It discovers hidden features of raw data by applying the representation-learning method. Deep Learning has the first layer as input layer, last layer as output layer and multiple hidden layers in between. Multilayer Perceptron (MLP), Convolutional Neural Networks (CNN), and Recurrent Neural Networks (RNN) are some of the deep learning architectures that are applied in fields, including image processing, machine translation, and sequence prediction. Multiple hidden layers signify the term “Deep” in “Deep Learning” [24]. Deep Learning does not have any global threshold of depth. According to Schmidhuber [25], deep learning has to have more than 10 hidden layers, though it is not a global threshold.

BRBES, as an expert system, makes reasoning through its rule base. However, it does not learn internal representation of external data independently. On the other hand, deep learning discovers hidden features from data of large volume. It does not have any rule base like an expert system. Driven by the power of deep learning for computing predictive output, we propose integrating Deep Learning with BRBES to increase the overall accuracy of the predictive output. Thus, our proposed integrated approach of BRBES and deep learning takes the research objective of developing a prediction model by combining the strength of both the systems through a novel mathematical model. To realize this objective, we address the following research questions in this paper:

(1) What is the benefit of applying BRBES to compute air pollution prediction?
  Better performance of BRBES than other knowledge-driven approaches in terms of dealing with uncertainties is the key benefit of applying BRBES over sensor data of air pollutants.
(2) What is the usefulness of adopting Deep Learning for air pollution prediction?
  Predicting pollution level based on the discovered hidden pattern of sensor data is the advantage of adopting Deep Learning architecture.
(3) Why and how to combine Deep Learning with BRBES?
  Improving accuracy of the prediction is the justification for integrating Deep Learning with BRBES.

We propose to achieve this integration through a novel mathematical model. We measure the concentration of PM_2.5_ by applying the Deep Learning technique on outdoor images through our proposed mathematical model. As a Deep Learning method, we use CNN to analyze the images. In case an image is hazy, we also evaluate whether this haze is caused by high PM_2.5_ or fog. At the same time, we take the PM_2.5_ reading of the same place directly from sensor device. Subsequently, we apply BRBES on both of these values to predict AQI while using our proposed novel algorithm. Thus, we achieve predictive output with higher accuracy by addressing uncertainty of sensor data while utilizing actionable insight that is gained from discovered data pattern.

The rest of the paper is organized, as follows: Section 2 presents the related works. In Section 3, we explain our proposed integrated approach of Belief Rule Base (BRB) and Deep Learning. In Section 4, we present our experimental results. Section 5 concludes the paper and presents our future research plans. 

## 2. Related Works 

Theang et al. [26] proposed Dynamically pre-trained Deep Recurrent Neural Network (DRNN) to predict the time-series level of PM_2.5_ in Japan. The network weights of this method are continuously updated to advance towards a dynamically and sequentially developing output, resulting in more precise learning representation of input data coming over time. Environmental monitoring data obtained from physical sensors have been used for this purpose. They have considered the spatial consistency of the concerned sensors to improve the prediction accuracy of DRNN. They have taken sensor reading of PM_2.5_ concentrations, wind speed, temperature, illuminance, humidity, and rainfall. They have discarded distant sensors with little impact to reduce the computational cost. They have screened out insignificant sensors by the elastic net method. DRNN’s prediction accuracy has turned out to be higher than the autoencoder training method. However, DRNN has not dealt with abnormal sensor data, which is likely to hamper prediction. 

Li et al. [27] has proposed Spatiotemporal deep learning (STDL) for predicting air pollution. It considers the spatiotemporal feature of sensor data. Stacked autoencoder (SAE) has been deployed as deep learning architecture to gain this feature. They have developed a regression model that is based on this learned representation. Their developed model can predict air quality of all stations simultaneously while ensuring temporal stability. They have applied their regression model on existing PM_2.5_ sensor data to predict the level of PM_2.5_. Their STDL model has shown higher accuracy than the spatiotemporal artificial neural network (STANN), auto regression moving average (ARMA), and support vector regression (SVR) models. However, this model also does not take into account uncertainty regarding the sensor data.

Kurt et al. [28] has employed Geographic Forecasting Models using the Neural Networks (GFM_NN) method to estimate the level of sulfur dioxide (SO_2_), carbon monoxide (CO), and particulate matter (PM_10_) of a Turkish district. They have fed the sensor data of 10 monitoring stations as input to feed-forward back-propagation neural network. They have come up with three geographic models for prediction purpose. The first model uses sensor data of a selected neighboring district. The second model takes two adjoining districts into account. The third model considers the distance between the triangulating districts and the target district. Their proposed geographic models have turned out to be more accurate than the non-geographic plain models. Their third geographic model, considering three districts, has performed better than other two models. Even though, GFM_NN has also left sensor data uncertainties unaddressed.

Moreover, Li et al. [29] presented a computer vision technique for assessing the haze level of images. They have estimated the transmission matrix of an input image with Dark Channel Prior (DCP) algorithm. Simultaneously, they have applied Deep Convolutional Neural Fields (DCNF) to estimate the depth map from pixels. The transmission matrix and depth map have been integrated through transformation functions. Subsequently, they piled up the matrix to a single figure with pooling function and determined haze level of an image. A combination of transmission and depth has resulted in haze level estimation with a higher accuracy than their separate application. The accuracy of their proposed method on PM_2.5_ dataset is 89.05% against 70.14% and 84.32% accuracy of only depth map and only transmission matrix, respectively. However, uncertainty that is associated with captured images is not dealt with by this method.

Liu et al. [30] has proposed an image-based approach, while considering several image features, such as transmission, sky smoothness, image color, entropy, contrast, time, geographical location, sun, and weather condition to predict the PM_2.5_ of the air. They have developed a regression model that is based on these features to predict PM level from photos of Beijing, Shanghai, and Phoenix over a one-year period. The inclusion of various image features has resulted in reasonable prediction accuracy of their method. The simplicity and smart phone readiness of this method can enable people to be more aware of air pollution. However, this model also has not considered uncertainty concerning the image data.

Zhan et al. proposed a standard haze image dataset [31] that contains haze images with all levels along with associated air quality data. Every image is tagged with the mean opinion score (MOS) as haze level’s subjective evaluation. They have also proposed a novel no-reference image quality assessment (IQA) method for assessing the haze quality of images by analyzing degradation-causing factors. IQA, when applied on this haze database, has shown promising results that are consistent with subjective evaluation. IQA has outperformed spatial and spectral entropies (SSEQ) and Blind/Referenceless Image Spatial Quality Evaluator (BRISQUE). Even though, IQA took no notice of image data uncertainty.

Table 1 illustrates the taxonomy of all of these air pollution prediction methods, in light of their strengths and limitations. Some of these approaches apply the neural network on numerical sensor data, while other methods adopt image processing algorithms. Hence, none of these works have processed both numerical and image sensor data concurrently. Neither did these works take sensor data uncertainties into account. Therefore, being stimulated by the efficacy of multimodal learning (as explained in Section 3.1), this research sheds light on the integrated processing of both numerical and image sensor data to improve the prediction accuracy while dealing with related uncertainties.

Neural network based Deep Learning methods can analyze images with reasonable accuracy. Three major classes of Deep Learning are: MLP, CNN, and RNN. MLP, which consists of more than one perceptron [32], can make non-linear classification. MLP’s learning approach is supervised [33]. It trains itself by a labeled dataset by discovering actionable insight. CNN is another class of Deep Learning, which is customized to analyze visual images. It resembles the structure of animal visual cortex [34,35]. Convolutional layers, pooling layers, fully connected layers, and normalization layers constitute CNN’s hidden layers [23,36]. Its learning approach is supervised, as it learns image features from labeled dataset. However, CNN cannot deal with uncertainty, such as camera crash, hardware problem, scratched glasses of a camera, ignorance, and obscure images [37]. Hence, such unexpected issues need to be dealt with to uphold prediction accuracy of CNN. RNN is also another class of artificial neural network. It is called recurrent because of its repetition of same process over all members of a sequence, where each predictive output is influenced by multiple previous observations [23]. RNN has its own memory, where it stores information concerning all of the calculations. This state retention capacity has made RNN suitable for tasks, such as predicting next word of a sequence and natural language processing. 

Problem domain of CNN and RNN is different from each other. CNN discovers spatial features and RNN retrieves temporal features. CNN is effective in processing high-dimensional images through its feature extraction characteristic. CNN can capture image features precisely through convolution operation, which is attributable to its higher depth than RNN. Therefore, it can be stated that CNN is the most appropriate Deep Learning class to analyze images with a view to predicting air pollution. 

## 3. Integrated Approach of BRB and Deep Learning

**Figure 1:**
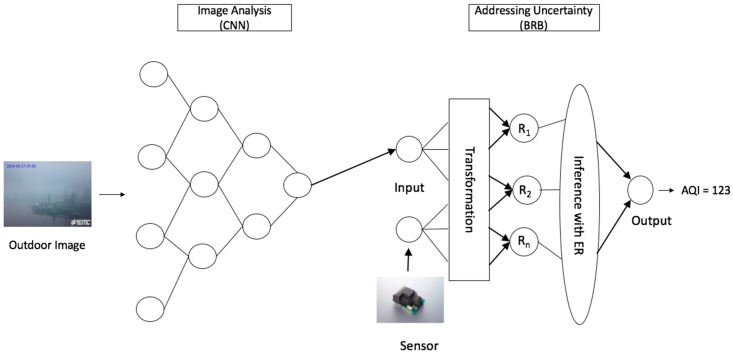
System Architecture.

Figure 1 shows the system architecture of our proposed integrated approach for predicting air pollution. We analyze outdoor images by Deep Learning method CNN to predict the concentration of PM_2.5_. Initially, CNN is trained by different outdoor images of the same place with a varying level of PM_2.5_. Thus, it learns the representation of an image’s PM_2.5_ level. Upon completion of the training, a new image of a certain time of the same place is fed to this trained CNN. Next, CNN performs analytics on this new image that is based on its training representation to predict the PM_2.5_ level of the concerned place. This CNN prediction output is recalculated if the haze of this image is caused by fog, rather than high PM_2.5_. This recalculated CNN output is then fed as input to BRBES. Further, numerical values of the level of PM_2.5_ of the same place and same time instance, being generated by the physical sensor device, are also fed to BRBES as input. Thus, BRBES has two input values with regard to PM_2.5_ level, one from CNN and the other from sensor reading. In this architecture, these two inputs constitute two antecedent attributes of BRBES. By reasoning over these two antecedent attributes, BRBES infers AQI as single numerical crisp value (as demonstrated in Section 3.3). Further, BRB calculates belief degree for each of the six AQI categories instead of demonstrating one single AQI category. Such a distributed assessment enables a person to have a holistic view of the overall environment (as demonstrated in Section 3.4). 

CNN has various architectures, such as, AlexNet, VGG Net, and GoogLeNet [38]. VGG Net has been adopted in this research due to its consistent architecture to extract image features. Researchers from Visual Geometry Group (VGG), University of Oxford, UK have presented VGG Net as CNN architecture [39]. It has total 19 layers, with 16 convolutional and 3 fully connected layers. It performs convolution operation over the input image matrix with small 3 × 3 receptive fields (stride 1) and extracts feature map. A deeper representation is advantageous for improving the classification accuracy. GoogLeNet, with 22 convolutional layers, has higher depth than VGG Net (16 convolutional layers). Even though, VGG Net’s network topology is simpler than GoogLeNet [40,41]. Moreover, GoogLeNet reduces feature maps’ spatial resolution at the beginning to decrease the computational cost. Conversely, AlexNet, with only five convolutional layers, has shallower depth than VGG Net. With respect to classification accuracy, VGG Net outperforms both GoogLeNet and AlexNet. Therefore, we have chosen VGG Net as our CNN model.

### 3.1. Rationale of Integration

Linking up information from various sources results in multimodal learning [42,43]. It discovers characterization over multiple modalities, rather than being reliant on one single modality. Being encouraged by the effectiveness of multimodal learning, we propose combining BRB and deep learning for improving AQI prediction accuracy. We take PM_2.5_ as our target air pollutant and calculate AQI with respect to this pollutant’s concentration. However, we consider both sensor data and image data (multiple modalities) to compute PM_2.5_ in addition to sensor data (single modality). Thus, we utilize the benefit of multimodal learning in this research. 

### 3.2. Neural Network Representation

This part applies deep learning to extract high-level representation from image. We have developed a smaller version of VGG Net for computing image-based PM_2.5_ prediction in this research.

**Figure 2:**
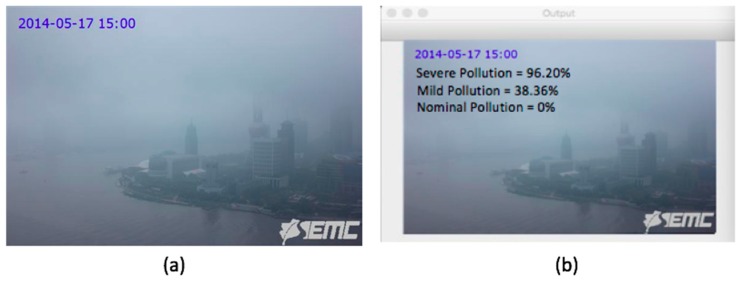
Image of Shanghai city, China captured by a camera on 17 May 2014 at 15:00 h: (**a**) original image captured by the camera; (**b**) VGG Net shows its predictive output label on the same image.

We have applied our mini VGG Net on air pollution images with volume 640 × 480 × 3 and 584 × 389 × 3. This VGG Net has five convolution layers and 1 fully connected layer. As activation function, we have used ReLU in this network. We have employed batch normalization to improve learning rate of our network. We have brought down overfitting of our model by adding 20% dropout to it. We have flattened the output of last pooling layer into a single vector. Our lone fully connected layer extracts 1024 features, after which 50% neurons are dropped. Our output layer, with softmax activation, calculates probability for three classes (Nominal Pollution, Mild Pollution, and Severe Pollution) through its three nodes. We have conducted 75 epochs over our VGG Net to discover image features through backpropagation. Our batch size has been set at 32, with 3982/32 = 125 iterations per epoch, where 3982 refers to the total number of training images. As optimization technique, we have used the Adam algorithm, which is an extended version of Stochastic Gradient Descent (SGD). Our VGG Net’s initial learning rate is Adam optimizer’s default value, 0.001. Table 2 demonstrates our VGG Net architecture. Multi-label binarizer has been applied to do multi-label image classification. For instance, VGG Net is run on a hazy image of Oriental Perl Tower, Shanghai, China captured by a camera sensor on May 17, 2014 at 15:00 hrs, as shown in Figure 2. It maps the probability of this hazy image for each of the three classes: severe, mild, and nominal pollution to 96.20%, 38.36%, and 0.00% respectively. These values are then normalized for uniformity in Equation (1).

Severe Pollution = 0.9620/(0.9620 + 0.3836 + 0.00) = 0.71 Mild Pollution = 0.3836/((0.9620 + 0.3836 + 0.00) = 0.29 Nominal Pollution = 0.00/((0.9620 + 0.3836 + 0.00) = 0.00(1)

Subsequently, we calculate PM_2.5_ by running Algorithm 1 over these three normalized values. This algorithm’s regression coefficients have been set in line with AQI breakpoint table. It returns PM_2.5_ concentrations as 188.97 µg/m^3^. However, this hazy image can be due to either high level of PM_2.5_ in the air or foggy weather. PM_2.5_ concentrations of 188.97 µg/m^3^ is practical if the haze level of the image is caused by suspended particulate matters in the air. On the other hand, these PM_2.5_ concentrations will be impractical if fog in the air causes this image to be haze, even though there is little to no PM_2.5_ in the air at that time instance. Hence, we propose Algorithm 2 to recalculate PM_2.5_ after confirming foggy weather. We only employ this algorithm if PM_2.5_ predicted by Algorithm 1 is more than or equal to 55.50. This 55.50 refers to the starting point of PM_2.5_ concentrations against the ‘Unhealthy’ AQI category. It has been done to ensure that only hazy images, rather than all images in general, pass through this Algorithm 2. In this algorithm, we calculate the Dew-point Temperature (DT) in degree celsius with a mathematical equation [44]. This equation uses the daily mean dry-bulb temperature (T) in degree celsius, and daily mean relative humidity (RH) in percentage. Fog forms in the air if the difference between instant air temperature and the Dew-point temperature is less than 2.5 degree celsius [45]. Therefore, in this algorithm, we check the difference between the instant air temperature (IT), when the photo was captured, and the Dew-point temperature (DT) of the site. We update the value of PM_Image, as calculated by Algorithm 1, if this difference is less than 2.5 degree celsius. In Line 3 of Algorithm 2, we divide the PM_2.5_ concentration (PM_CNN), as predicted by Algorithm 1, by 500.4, which, according to the EPA breakpoint table, is the highest 24-hour concentration of PM_2.5_. In the next line, we update the value of PM_Image by multiplying the division result with 55.40. This 55.40 refers to the average PM_2.5_ concentration of 55.40 µg/m^3^ if the relative humidity of the air is above 87% [46].
**Algorithm 1:** an algorithm to achieve image based prediction of PM_2.5_
**Input:** SP denotes the normalized probability that the image belongs to Severe Pollution class; MP denotes the normalized probability of Mild Pollution class, and NP denotes the normalized probability of Nominal Pollution class. **Output:** PM_2.5_ concentrations predicted from the image (PM_Image). Begin1 **if** ((SP > MP) **and** (SP > NP)) **then**2 PM_Image = (150.5 + 275.9* SP) + (150.4 * MP)/23 **else if** ((NP > SP) **and** (NP > MP)) **then**4 PM_Image = (35.4 * (1 – NP)) + ((150.4 * MP)/2)5 **else if** ((MP > SP) **and** (MP > NP)) **then**
6 **if** (SP > NP) **then**7  PM_Image = (35.5 + 114.9 * MP) + ((500.4 * SP)/2) 8 **else if** (NP > SP) **then**9  PM_Image = (35.5 + 114.9 * MP) + ((35.4 * NP)/2) 10 **return** PM_Image End 

Temperature of the Shanghai site from where the image was taken (Figure 2) is 18 degree celsius on May 17, 2014 at 15:00 h, according to China’s Shanghai city weather dataset (as explained in Section 4.1.2). Dew-point temperature at the same place and time is 16 degree celsius. As difference between instant temperature and dew-point temperature is (18 - 16) or 2 degree celsius, Algorithm 2 confirms the presence of fog. Hence, this algorithm recalculates the PM_2.5_ concentration to be 20.92 µg/m^3^, which was initially predicted to be 188.97 µg/m^3^ by Algorithm 1. Thus, Algorithm 2 rectifies erroneous PM_2.5_ concentration predicted from image, in the case the haze of the image is caused by foggy weather, rather than particulate matters that are suspended in the air.
**Algorithm 2:** an algorithm to recalculate PM_2.5_ in a foggy weather **Input:** T denotes the daily mean dry-bulb temperature (in degree celsius); RH denotes the instant relative humidity (between 0 and 1); IT denotes the instant on-site temperature (in degree celsius), and PM_CNN denotes the PM_2.5_ concentrations predicted by CNN in Algorithm 1. **Output:** PM_2.5_ concentration in case the weather is foggy (PM_Image). Begin 1 **if** ((PM_CNN) >= 55.50)2 DT = T – ((100-RH)/5)3 **if** ((IT- DT) < 2.5) 4  PO = (PM_CNN)/500.45   PM_Image = 55.40 * PO6 **return** PM_Image End 

### 3.3. Integration of CNN with BRBES 

This section explains the functional system of BRBES and its integration with CNN. The reasoning approach of BRBES consists of four steps—Input transformation, Rule activation weight calculation, Belief degree update, and the Rule aggregation [47].

#### 3.3.1. Domain Knowledge Representation

A belief rule consists of two portions: the antecedent part and consequent part. The antecedent part has several antecedent attributes with referential values. The consequent part has one single consequent attribute with its own referential values. We have two antecedent attributes in our rule base: sensor reading of PM_2.5_ and image-based PM_2.5_ prediction. Each of the antecedent attributes has three referential values: High, Medium, and Low. For instance, a certain rule is formulated as reading of PM_2.5_ and image-based PM_2.5_ prediction. Each of the antecedent attributes has three referential values: High, Medium, and Low. For instance, a certain rule is formulated as

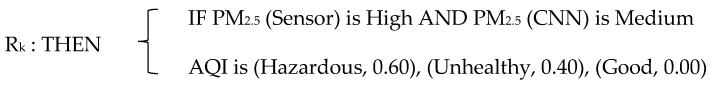

where R_k_ indicates the kth rule of our rule base and {(Hazardous, 0.60), (Unhealthy, 0.40), and (Good, 0.00)} represents the (referential value, belief degree) pair of the three referential values of the consequent attribute ‘AQI’. We have developed the rule base of BRB part based on the AQI breakpoint table of EPA [12]. We demonstrate all nine rules of our rule base in Table 3. The activation weight’ column of this table has been explained later in this subsection. The reasoning system of BRBES is presented below. 

#### 3.3.2. BRBES Reasoning System 

##### Input Transformation

Sensor reading is distributed into its referential values in this stage. We have set the utility values for “Low”, “Medium”, and “High” are h_i1_ = 0, h_i2_ = 150.5, and h_i3_ = 500.4, respectively. Procedure of input transformation is as follows:

IF h_i3_ >= input >= h_i2_ THEN Medium = (h_i3_ − input)/(h_i3_ − h_i2_)

High = (1 − Medium), Low = 1 − Medium − High

IF hi_2_ > input >= hi_1_ THEN Low = (hi_2_ − input)/(h_i2_ − h_i1_)

Medium = (1 − Low), High = 1 − Low − Medium

According to Shanghai weather dataset (as explained in Section 4.1.2), the sensor reading of PM_2.5_ of the Shanghai site (as shown in Figure 2) is 35 µg/m^3^ on May 14, 2017 at 15:00 h. We transform it into its referential values.

Low_Sensor, L1 = (150.5 − 35)/(150.5 − 0) = 0.77;
Medium_Sensor, M1 = (1 − 0.77) = 0.23 and
High_Sensor, H1 = (1 − 0.77 − 0.23) = 0.

We then transform the PM_2.5_ concentrations 20.92 µg/m^3^ predicted from image (by Algorithm 1 and Algorithm 2) into its referential values, as follows.

Low_CNN, L2 = (150.5 − 20.92)/(150.5 − 0) = 0.86;
Medium_CNN, M2 = (1 − 0.86) = 0.14 and
High_CNN, H2 = (1 − 0.86 − 0.14) = 0.0

##### Rule Activation Weight Calculation

This part requires the referential value’s matching degree at which the belief is matched [18]. The matching degree of kth rule is:

(2) $$\alpha_{k}=\overset{T_{k}}{\underset{i=1}{\prod }}{\left(\alpha_{i}^{k}\right)}^{\bar{\delta_{\mathrm{ki}}}}{\mathrm{and}\;\alpha }_{k}=\overset{T_{k}}{\underset{i=1}{\prod }}{\left(\alpha_{i}^{k}\right)}^{\bar{\delta_{\mathrm{ki}}}}$$

where T_k_ refers to kth rule’s total number of antecedent attributes and δ_ki_ is i^th^ antecedent attribute’s weight. Matching degrees are assigned to the referential values of the antecedent attributes to activate a rule [47]. The activation weight of k^th^ activated rule is defined as:

(3) $$\omega_{k}=\frac{\theta_{k\;}\alpha_{k}}{\sum_{j=1}^{L}\theta_{j}\alpha_{j}}=\frac{\theta_{k}\prod_{i=1}^{T_{k}}{\left(\alpha_{i}^{k}\right)}^{\bar{\delta_{\mathrm{ki}}}}}{\sum_{j=1}^{L}\theta_{j}\prod_{i=1}^{T_{k}}{\left(\alpha_{i}^{k}\right)}^{\bar{\delta_{\mathrm{ki}}}}}$$

where $\bar{\_\mathrm{ki}}$ refers to the i^th^ antecedent attribute’s relative weight in the k^th^ rule and θ_k_ is the rule weight of k^th^ rule. The value of θ_k_ is between 0 and 1 (both inclusive). The activation weight of each of the nine rules, as calculated using Equation (3), has been shown in the last column of Table 3.

##### Belief Degree Update

Sensor data becomes unavailable in the case of uncertainty due to ignorance. For example, suddenly, we might lack the sensor data against one of the antecedent attributes of our system. To address such exceptional cases, we update the initial belief degrees of the referential values of the consequent attribute with a mathematical equation [47].

##### Rules Aggregation 

The ER mechanism, either recursive or analytical, is employed to aggregate the rules of BRBES [14]. However, the analytical ER approach is computationally less complex than its recursive counterpart [48]. Therefore, we have calculated the belief degree of all the referential values of consequent attribute with analytical ER. The final result C(Y) is defined as:

C(Y) = {(O_j_, β_j_), j = 1, …, N} (4)

where β_j_ refers to the belief degree of referential value O_j_ of the consequent attribute, which has N number of referential values. β_j_, belief degree of consequent attribute’s concerned referential value, is defined as:

(5) $$\beta_{j}=\frac{\mu \times \left[\prod_{k=1}^{L}\left(\omega_{k}\beta_{\mathrm{jk}}+1-\omega_{k}\sum_{j=1}^{N}\beta_{\mathrm{jk}}\right)-\prod_{k=1}^{L}\left(1-\omega_{k}\sum_{j=1}^{N}\beta_{\mathrm{jk}}\right)\right]}{1-\mu \times \left[\prod_{k=1}^{L}\left(1-\omega_{k}\right)\right]}$$

where L is the number of rules in rule base and μ is defined as:

(6) $$\mu ={\left[\overset{N}{\underset{j=1}{\sum }}\overset{L}{\underset{k=1}{\prod }}\left(\omega_{k}\beta_{\mathrm{jk}}+1-\omega_{k}\overset{N}{\underset{j=1}{\sum }}\beta_{\mathrm{jk}}\right)-\left(N-1\right)\overset{L}{\underset{k=1}{\prod }}\left(1-\omega_{k}\overset{N}{\underset{j=1}{\sum }}\beta_{\mathrm{jk}}\right)\right]}^{-1}$$

Our calculated belief degrees of all the referential values of consequent attribute while using Equation (5) are as follows:

Good = 0.9098; Unhealthy = 0.0902 and Hazardous = 0.0

**Figure 3:**
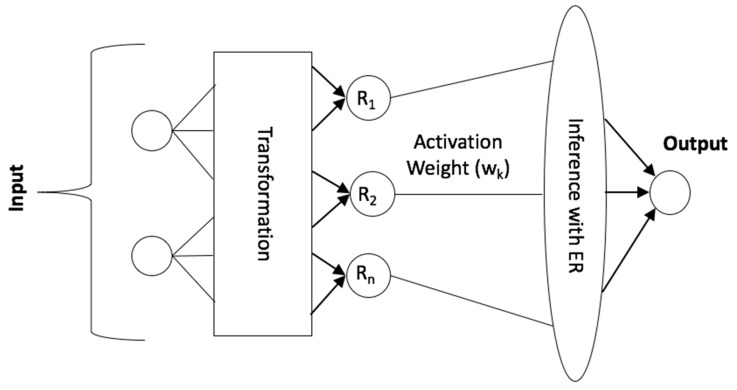
Conceptual architecture of Belief Rule Based Expert System (BRBES).

Subsequently, we employ Algorithm 3 to transform this multi-value assessment into one single numerical crisp value. This algorithm has resulted in the crisp value of AQI as: ((100 × (1 − 0. 9098)) + ((200 × 0. 0902)/2)) = 18.04. Figure 3 shows the conceptual architecture of BRBES.
**Algorithm 3:** an algorithm to calculate single numerical crisp value of AQI **Input:** H denotes the belief degree of the referential value ‘Hazardous’ of the consequent attribute ‘AQI’; U denotes the belief degree of ‘Unhealthy’ referential value, and G denotes the belief degree of ‘Good’ referential value of the consequent attribute. **Output:** The crisp value of AQI (Q). Begin1 **if** ((H > U) **and** (H > G)) **then**2 Q = (201 + 299*H) + ((200*U)/2)3 **else if** ((G > H) **and** (G > U)) **then**4 Q = (100*(1 − G)) + ((200*U)/2)5 **else if** ((U > H) **and** (U > G)) **then**6 **if** (H > G) **then**7  Q = (101 + 99*U) + ((500*H)/2) 8 **else if** (G > H) **then**
9  Q = (101 + 99*U) + ((100*G)/2)10 **return** Q End 

#### 3.3.3. Disjunctive BRBES

The rule base of BRBES that we have demonstrated in Section 3.3.1 is of conjunctive type. Such conjunctive rule base is constructed based on every possible combination of referential values of the antecedent attributes [49]. Thus, consumptive assumption creates a large rule base, in case the number of referential values and/or antecedent attributes is too high. Hence, the memory and computational requirement of this assumption is high. Researchers have come up with disjunctive BRBES to address this shortcoming [49,50]. The number of referential values of all antecedent attributes is equal in disjunctive BRB [51]. This BRB has the same number of rules, as the number of referential values of its antecedent attributes. Disjunctive BRB, having a small rule base, calculates the activation weight of its k^th^ rule, w_k_, as

(7) $$w_{k}=\frac{\theta_{k}\sum_{i=1}^{M}\alpha_{i}^{k}}{\sum_{l=1}^{L}\theta_{l}\sum_{i=1}^{M}\alpha_{i}^{l}}$$

where θ_k_ is the initial weight of the k^th^ rule, α_i_^k^ refers to the input matching degree with k^th^ rule, and L and M refers to the total number of rules and inputs, respectively. Disjunctive BRB activates a rule, even with one non-zero input matching degree. On the contrary, conjunctive BRB does not activate a rule, even if one input matching degree is 0. Thus, disjunctive BRB reduces memory capacity and computational cost by bringing down the size of rule base. We show the rule base of our system, constructed under disjunctive assumption, in Table 4.

#### 3.3.4. Joint Optimization of BRBES

There are two ways to optimize the performance of BRBES: a) Parameter Optimization and b) Structure Optimization. The combined application of both of these techniques results in joint optimization.

The parameters we have considered for optimization are: attribute weight, rule weight, and consequent part’s belief degrees. We have applied Differential Evolution (DE) to perform parameter optimization [52]. We also have run BRB adaptive DE (BRBaDE) to adjust the values of two control parameters of DE: Crossover Factor (CR) and Mutation Factor (F). BRBaDE hits a balance between exploration and exploitation while setting proper values of these control parameters [53]. We execute Structure Optimization that is based on the Heuristic Strategy (SOHS) algorithm to perform structure optimization of BRBES [54]. SOHS makes comparative analysis of prediction accuracy of BRBES with varying number of referential values of the antecedent attributes and selects the one with the lowest error. We achieve joint optimization through Joint Optimization on Parameter and Structure (JOPS) algorithm [54]. JOPS runs SOHS over a set of DE-optimized BRBESs. Again, DE is applied on the SOHS optimized set of BRBESs until the stop criterion is satisfied. Finally, JOPS selects the BRBES with the lowest error as its output.

**Figure 4:**
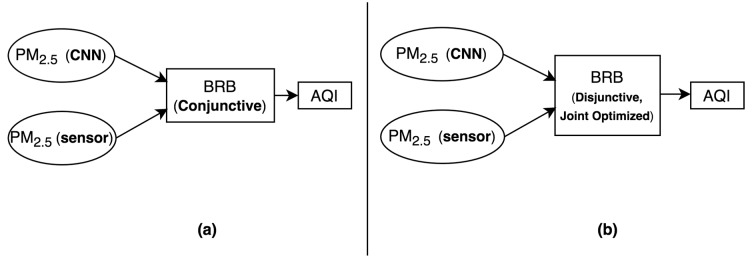
General methodological scheme of our proposed system: (**a**) with Conjunctive Belief Rule Base (BRB); (**b**) with Disjunctive, Joint Optimized BRB.

Figure 4 shows the general methodological scheme of our proposed system. In Figure 4a, conjunctive BRB receives PM_2.5_ concentrations as its input both from CNN and sensor device. It performs reasoning over these two input values to infer AQI as its output. Figure 4b also infers AQI with respect to PM_2.5_ concentrations that are computed by CNN and sensor device. However, in Figure 4b, disjunctive and joint optimized BRB, instead of the conjunctive one as in Figure 4a, has been used to make this AQI inference. Thus, Figure 4b comes up with a more memory efficient and accurate version of Figure 4a.

### 3.4. Distributed Categorization of AQI

This part distributes belief degrees to all six AQI categories via a regression layer. We apply a regression coefficient for this purpose. We calculate the belief degree of the c^th^ AQI category, as:

ÿ_ic_ = categorize(aqi_predicted ) = r_c_ * b_c_ (8)

where r_c_ is a regression coefficient and b_c_ is the belief degree of relevant referential value of the consequent attribute. We show the distributed belief degrees of all the six AQI categories as ÿ_i_ = [ÿ_i1_, ÿ_i2_, … ÿ_i6_]. We calculate r_c_ based on the predicted crisp value of AQI by employing Algorithm 4. This algorithm calculates r_c_ to be 18.04/49 = 0.3682. Finally, the belief degrees of all six AQI categories are:

Belief Degree for ‘Good’ category, ÿ_i1_ = (Good) * (1 − r_c_)
Belief Degree for ‘Moderate’ category, ÿ_i2_ = (Good) * r_c_
Belief Degree for ‘Unhealthy for sensitive groups’ category, ÿ_i3_ = (Unhealthy) * (1 − r_c_)
Belief Degree for ‘Unhealthy’ category, ÿ_i4_ = (Unhealthy) * r_c_
Belief Degree for ‘Very Unhealthy’ category, ÿ_i5_ = (Hazardous) * (1 − r_c_)
Belief Degree for ‘Hazardous’ category, ÿ_i6_ = (Hazardous) * r_c_

Here, ‘Hazardous’, ‘Unhealthy’, and ‘Good’ refer to the belief degree of the concerned referential value. Now, we calculate the distributed belief degrees of each of the six AQI categories.

Belief Degree for ‘Good’ category, ÿ_i1_ = (0.9098) * (1 − 0.3682) = 0.58
Belief Degree for ‘Moderate’ category, ÿ_i2_ = (0.9098) * 0.3682 = 0.33
Belief Degree for ‘Unhealthy for sensitive groups’ category, ÿ_i3_ = (0.0902) * (1 − 0.3682) = 0.06
Belief Degree for ‘Unhealthy’ category, ÿ_i4_ = (0. 0902) * 0.3682 = 0.03
Belief Degree for ‘Very Unhealthy’ category, ÿ_i5_ = (0.0) * (1 − 0.3682) = 0
Belief Degree for ‘Hazardous’ category, ÿ_i6_ = (0.0) * 0.3682 = 0

Subsequently, we calculate the Mean Square Error, E of our proposed system with respect to the difference between predicted and actual AQI over m training data pairs, as defined in Equation (9).

(9) $$E\;=\frac{1}{m}{\_}_{i=0}^{m-1}{\left(\mathrm{aqi}\_\mathrm{predicted}-\mathrm{aqi}\_\mathrm{actual}\right)}^{2}\;$$

Here, aqi_predicted means the crisp value of AQI and aqi_actual is the one from dataset.
**Algorithm 4:** an algorithm to calculate regression coefficient based on predicted AQI crisp value **Input:** Q denotes the predicted crisp value of AQI.**Output:** The regression coefficient (r_c_). Begin1 **if** (Q >= 301) **then**2 r_c_ = (Q - 301)/1993 **else if** ((Q >= 201) **and** (Q <= 300)) **then**4 r_c_ = (Q - 201)/99 5 **else if** ((Q >= 151) **and** (Q <= 200)) **then**6 r_c_ = (Q - 151)/49 7 **else if** ((Q >= 101) **and** (Q <= 150)) **then**8 r_c_ = (Q - 101)/49 9 **else if** ((Q >= 51) **and** (Q <= 100)) **then**10 r_c_ = (Q - 51)/49 11 **else if** (Q <= 50) **then**
12 r_c_ = Q /49 13 **return** r_c_
End 

## 4. Experiments

We have used Python 3.6.4 and Keras neural network library for implementation purpose. We have developed both BRBES and CNN with the python programming language. As an image processing library, we have used OpenCV to process the air pollution images. We have used Keras library functions to implement our VGG Net. Prediction accuracy of our VGG Net over the testing part of air pollution images has turned out to be around 87.78%. We have fed VGG Net’s multi-value predictive output to BRB script through standard file I/O.

### 4.1. Dataset

We have applied our proposed predictive algorithm on two different datasets. One is a labeled dataset of synthetic images with corresponding PM_2.5_ label. The other one consists of both hourly real images of China’s Shanghai city and corresponding PM_2.5_ concentrations as well as other relevant meteorological data.

#### 4.1.1. Synthetic Image Dataset

We have used the labeled dataset of air pollution images that was provided by Li et al. [29]. The dimensions of these images are 640 × 480, with RGB color space. This dataset contains air pollution images along with corresponding numerical value of PM_2.5_ concentrations. These are synthetic images which have been developed artificially to reflect various levels of PM_2.5_ concentrations. These are not captured from any place on the earth. Hence, these images do not have any temporal resolution, such as hourly or daily images. This dataset does not require any sensor device, as both image and corresponding PM_2.5_ concentrations are artificially generated.

**Figure 5:**
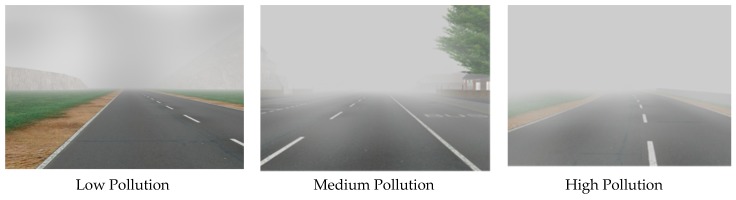
Synthetic Air Pollution images with low, medium and high levels.

This dataset has 3024 synthetic images with a varying level of air pollution. Each image is tagged with PM_2.5_ level of the same place and same time. We have bifurcated these 3024 images into two parts: 2419 training images and 605 testing images. We use training images to train up our VGG Net. Subsequently, we evaluate this network’s reliability with testing images. We have divided these 2419 training images into three parts: High Pollution, Medium Pollution, and Low Pollution. Here, High refers to 150.5 µg/m^3^ and a higher value of PM_2.5_, Medium refers to 35.5 to 150.4 µg/m^3^ of PM_2.5_, and Low refers to PM_2.5_ below 35.5 µg/m^3^. We show three sample training images from the dataset in Figure 5.

#### 4.1.2. Shanghai City Dataset

This dataset contains 1954 real images, with dimensions 584 x 389 and RGB color space, of the Oriental Pearl Tower, Shanghai, China [30]. These images, which were collected from Archive of Many Outdoor Scenes (AOMS) dataset, are hourly images that are captured every hour from 08:00 to 16:00 hrs during May to December in 2014 [55]. Moreover, we have included hourly sensor reading of PM_2.5_ concentrations and few other weather data of Shanghai covering 00:00 to 23:00 hrs from May to December 2014. These PM_2.5_ readings and numerical weather data are obtained from Liang et al. [56]. Their provided dataset contains PM_2.5_ reading from three different sources: one from the U.S. consulate in Shanghai and the other two are from two neighboring sites of China’s Ministry of Environmental Protection (MEP) in Shanghai. These two MEP sites are located in the Jingan and Xuhui districts of Shanghai. The distance of Jingan and Xuhui to the U.S. consulate in Shanghai is 2.5 km and 5 km, respectively. We have included PM_2.5_ reading from U.S. consulate in our dataset. In case the U.S. consulate reading is unavailable for a certain time instance, we have considered Jingan site reading. In terms of Jingan site data unavailability, Xuhui site data have been taken into account. Jingan has been preferred to Xuhui due to Jingan’s higher proximity to the U.S. consulate than Xuhui. Moreover, we have incorporated Shanghai weather data into our dataset to evaluate whether the weather was foggy when an image was captured. These weather data, which were recorded at the Shanghai Airport, contain hourly measurements of temperature, relative humidity, dew point, pressure, wind direction and speed, and precipitation. Among these parameters, temperature, dew point, and relative humidity have been used in Algorithm 2 in order to assess foggy weather. The high haze level of the Shanghai image, as shown in Figure 2, is not due to high PM_2.5_, but because of fog. We have determined this by taking the difference between temperature and dew point when that image was captured into account. Thus, Shanghai weather data play a significant role in this research in distinguishing a hazy image between polluted air and foggy weather.

**Figure 6:**
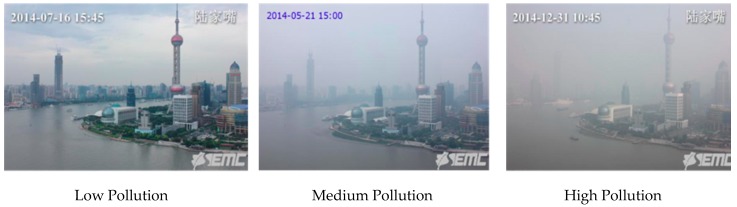
Shanghai Air Pollution images with low, medium, and high levels.

We have divided these 1954 Shanghai images into two parts: 1563 training images and 391 testing images. We have also split these 1563 training images into three parts: High Pollution, Medium Pollution, and Low Pollution. The PM_2.5_ range against High, Medium, and Low Pollution is the same as mentioned in Section 4.1.1. Figure 6 illustrates three sample training images of Oriental Pearl Tower, Shanghai with low, medium, and high level of air pollution.

### 4.2. Results

We demonstrate the lower error of our proposed system than other approaches in Table 5 when the sensor gives a wrong reading of PM_2.5_ due to technical malfunction. For instance, in terms of the wrong sensor reading of 126 µg/m^3^, against an accurate reading of 447 µg/m^3^, our proposed approach’s AQI (263.96) is closer to the actual AQI (464) when compared to the one only predicted by BRB (208.34).

**Figure 7:**
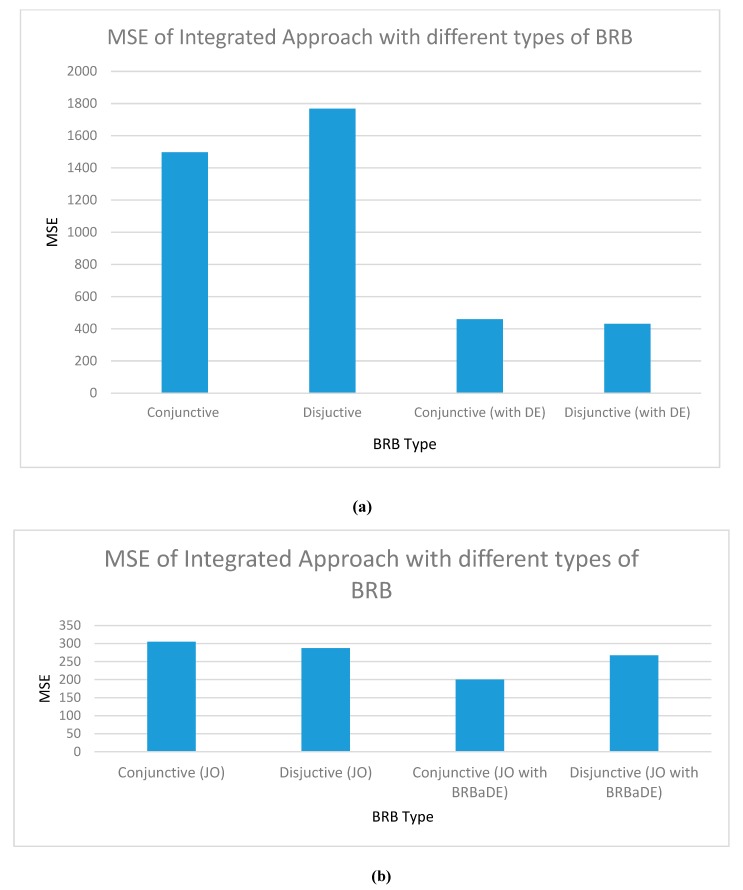
Testing dataset Mean Square Error (MSE) of our integrated approach with Conjunctive and Disjunctive BRB: (**a**) non-trained and DE-optimized; (**b**) Joint Optimized (JO), including BRB adaptive DE (BRBaDE).

Figure 7a shows a higher Mean Square Error (MSE) of disjunctive BRB than its conjunctive counterpart. Here, MSE refers to the gap between actual AQI (collected from the dataset) and predicted AQI (as predicted by our proposed model). Disjunctive assumption’s lesser amount of reasoning because of its small rule base has resulted in this higher error. In terms of the DE-led parameter optimized mode, disjunctive BRB offers higher accuracy than the conjunctive one, as shown in Figure 7b. DE optimizes 17 parameters of disjunctive BRB, against 41 parameters in conjunctive assumption. Dealing with less number of parameters in disjunctive assumption has resulted in its higher accuracy. In terms of structure optimization, the optimum number of referential values of antecedent attributes for conjunctive and disjunctive BRB has turned out to be three and four, respectively. Finally, in a jointly optimized state, Figure 7b shows a higher accuracy of disjunctive BRB than conjunctive one. During joint optimization, DE only fine-tunes 32 parameters in disjunctive assumption, against 152 of conjunctive BRB. The higher accuracy of joint optimized disjunctive BRB is attributed to adjustment of lower number of parameters than the conjunctive one. In Figure 7b, we also show the lower error of conjunctive BRB than the disjunctive one, when DE is replaced with BRBaDE in JOPS. BRBaDE positively impacts more than double parameters in conjunctive BRB than the disjunctive one, which results in conjunctive assumption’s higher performance.

**Figure 8:**
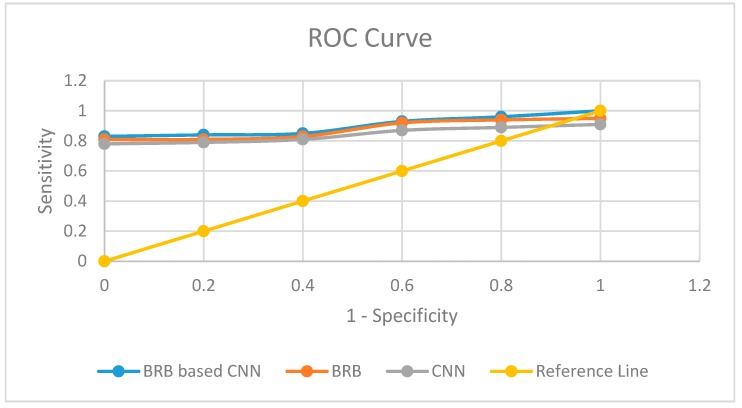
Comparison of results with Receiver Operating Characteristic (ROC) curves.

Different machine learning techniques, such as, Random Forest, Decision Tree, ANN, and Linear Regression are outperformed by BRBES in terms of prediction accuracy [57]. Hence, we perform comparative analysis of our integrated approach with only BRB (conjunctive, non-trained) and only CNN. We have taken joint optimized disjunctive assumption as BRB part of our integrated approach. Table 6 shows the sensitivity, specificity, and Area Under Curve (AUC) of each of the three techniques. The higher accuracy of our integrated system than other two approaches is attributable to the adoption of multimodal learning. We have employed the Receiver Operating Characteristic (ROC) curve to make the visualization of comparative performance of these three predictive models [58]. We show the ROC of these three models in Figure 8. A higher value of AUC refers to more reliability of a predictive model. Table 6 shows our proposed system’s higher AUC, sensitivity, and specificity. Therefore, it can be stated that our integrated model is dependable enough to make AQI prediction with rational accuracy.

**Figure 9:**
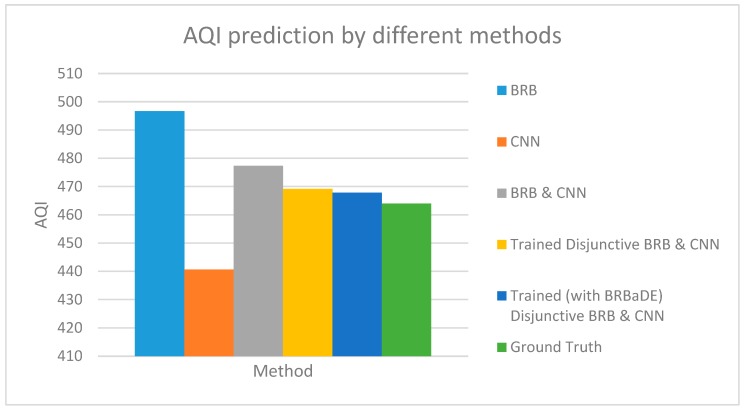
Air Quality Index (AQI) prediction by BRB, Convolutional Neural Networks (CNN), and our proposed integrated approach (BRB and CNN).

**Figure 10:**
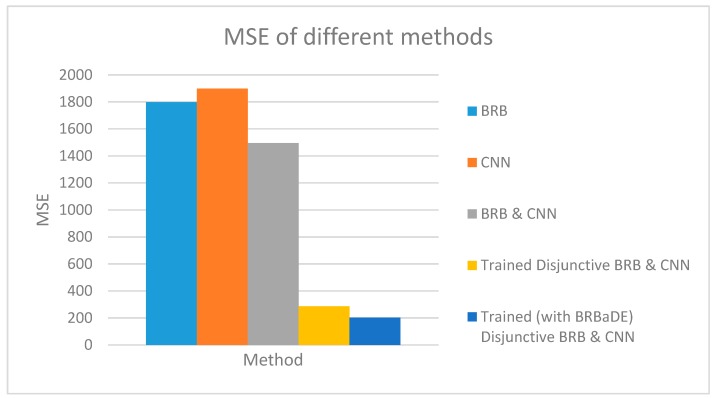
Testing dataset MSE of BRB, CNN, and our proposed integrated approach (BRB and CNN).

We predict AQI with our proposed approach, only BRB and only CNN based on an example sensor reading of 464 µg/m^3^. Our proposed system has been employed with non-trained conjunctive BRB and trained disjunctive BRB (with DE and BRBaDE), as shown in Figure 9. AQI closest to the ground truth has been computed by our proposed system with trained disjunctive BRB. We also show the testing dataset MSE of these five methods in Figure 10, with our proposed approach having the lowest MSE. Thus, we rationalize the adoption of multimodal learning as well as the trained version of BRB in this research.

### 4.3. Discussion

From the results demonstrated in Section 4.2, it is clearly evident that our proposed integrated approach performs better than only BRB, only CNN, as well as other machine learning techniques. A higher prediction accuracy of our proposed integrated system becomes more evident when the sensor computes wrong reading of PM_2.5_ concentrations or CNN predicts high PM_2.5_ from a hazy image, even though haze is triggered by fog, rather than polluted air. We improve the efficiency of our system, in terms of computational cost and prediction accuracy, by incorporating disjunctive assumption and joint optimization into BRB part of our proposed approach. Moreover, we show even higher prediction accuracy while performing parameter optimization with BRBaDE, instead of DE.

## 5. Conclusions

This study presented a BRB based Deep Learning system as a novel predictive analytics technique for predicting the level of air pollution in terms of AQI. We clearly rationalized the choice of VGG Net over other CNN architectures. We examined various shortcomings of prevailing air pollution prediction system and addressed those drawbacks by our proposed approach. Such a prediction enables the authorities and citizens to be warned of air pollution in advance and take appropriate precautionary measures. Further, an efficient AQI prediction model evaluates the sensitivity of public health with respect to air quality. Hence, the accurate prediction of air pollution plays a strong role to make the world more sustainable. We used the labeled dataset of synthetic images as well as real-world images and weather data of Shanghai to demonstrate the higher efficiency of our proposed model. The integration of Deep Learning with BRB through a novel mathematical model has resulted in this improved accuracy. We also distinguished a hazy image between foggy weather and actually polluted air through our proposed predictive algorithm. We then employed a disjunctive assumption of BRB to make our system more efficient in terms of computational cost and memory requirement. Moreover, we applied joint optimization to fine-tune the learning parameters and structure of BRB. Such optimization techniques have contributed to a significant increase of the AQI prediction accuracy of our proposed integrated system. We implemented our prediction system in python language. The results showed that our optimized integrated approach outperformed only BRB and only CNN, which is attributed to the combined utilization of BRBES’s uncertainty handling capacity and CNN’s data pattern discovery. Our integrated model has the flexibility to be applied on various other application areas of sensor data streams to infer a predictive output. Such areas include predictive maintenance, flu pattern prediction, data center energy consumption prediction and so on. In short, this study demonstrated the power of accuracy to achieve predictive output.

In the future, we plan to evaluate our model performance by dataset amounting to petabytes or yottabytes with Hadoop ecosystem. Predicting PM_2.5_ directly from satellite images, rather than ground images, is also part of our future research works. We also intend to incorporate real-time validation in the future to evaluate the consistency of our system on real-time basis while using a real-world dataset of images and PM_2.5_ concentrations. Moreover, incorporating deterministic air quality model into our proposed system, through combination of relevant data sources, to accurately observe long-term air pollution trend of a certain geographical area, constitutes part of our future research direction.

## Figures and Tables

**Table 1:** Taxonomy of related works.

Article	Specification	Method	Limitation
[26]	This prediction model predicts time-series concentrations of PM_2.5_ in Japan. In addition to time, it considers physical position of sensors to improve prediction accuracy.	Deep Recurrent Neural Network (DRNN)	This model applies Deep Learning on sensor data. However, it does not consider uncertainties associated with such sensor data.
[27]	This system predicts PM_2.5_ level based on available PM_2.5_ sensor data. It considers interrelationship between space and time concerning the sensor reading.	Spatiotemporal deep learning (STDL)	This system applies deep learning architecture to learn spatiotemporal features of sensor data. However, it does not deal with uncertainties of sensor data.
[28]	This method predicts the level of SO_2_, CO and PM_10_ of a Turkish district. It’s distance-based geographic model offers higher accuracy than non-geographic model.	Geographic Forecasting Models using Neural Networks (GFM_NN)	This method considers sensor data of neighboring district as well as distance between neighboring and target district to improve prediction accuracy. Still, it does not address sensor data uncertainties, which are likely to hamper prediction accuracy.
[29]	It is an image-based method to evaluate haze level of images. It has combined transmission matrix and depth map of images and demonstrated its higher accuracy on PM_2.5_ dataset.	Combined application of Dark Channel Prior (DCP) and Deep Convolutional Neural Fields (DCNF)	This method has demonstrated higher accuracy than separate application of transmission matrix and depth map. However, the uncertainty associated with image data is left unaddressed.
[30]	This paper presents a regression model to predict PM_2.5_ level from images of Beijing, Shanghai and Phoenix. It has considered various image features as part of this process.	Support Vector Regression (SVR)	Consideration of various image features has made this model quite representative. Even though, uncertainty handling of captured images is disregarded.
[31]	This paper proposes a haze image dataset with weather information. It presents an image based technique to evaluate the haze images.	Image Quality Assessment (IQA)	This IQA technique can properly assess the haze level of images. However, it disregards the uncertainties of images taken by camera sensor.

**Table 2:** Visual Geometry Group (VGG) Net Architecture.

Model Content	Details
Input image size	640 × 480 × 3, 584 × 389 × 3
First Convolution Layer	32 filters of size 3 × 3, ReLU,
First Max Pooling Layer	Pooling Size 3 × 3
Second Convolution Layer	64 filters of size 3 × 3, ReLU
Second Max Pooling Layer	Pooling size 2 × 2
Third Convolution Layer	64 filters of size 3 × 3, ReLU
Third Max Pooling Layer	Pooling size 2 × 2
Fourth Convolution Layer	128 filters of size 3 × 3, ReLU
Fourth Max Pooling Layer	Pooling size 2 × 2
Fifth Convolution Layer	128 filters of size 3 × 3, ReLU
Fifth Max Pooling Layer	Pooling size 2 × 2
Fully Connected Layer	1024 nodes, ReLU
Dropout Layer	excludes 50% neurons randomly
Output Layer	3 nodes for 3 classes, SoftMax
Optimization Function	Adam optimization algorithm
Learning Rate	0.001
Loss Function	Binary Cross Entropy

**Table 3:** Initial Rule Base.

Rule Id	Rule Weight	IF		THEN			Activation Weight
		PM_2.5_ (Sensor)	PM_2.5_ (CNN)	AQI			
				Hazardous	Unhealthy	Good	
R1	1.0	H	H	1.00	0.00	0.00	0.58
R2	1.0	H	M	0.60	0.40	0.00	0.25
R3	1.0	H	L	0.60	0.20	0.20	0.00
R4	1.0	M	H	0.40	0.60	0.00	0.12
R5	1.0	M	M	0.00	1.00	0.00	0.05
R6	1.0	M	L	0.00	0.60	0.40	0.00
R7	1.0	L	H	0.20	0.20	0.60	0.00
R8	1.0	L	M	0.00	0.40	0.60	0.00
R9	1.0	L	L	0.00	0.00	1.00	0.00

**Table 4:** Rule base under disjunctive assumption.

Rule Id	Rule Weight	IF		THEN		
		PM_2.5_ (Sensor)	PM_2.5_ (CNN)	AQI		
				Hazardous	Unhealthy	Good
R1	1.0	H	H	1.00	0.00	0.00
R2	1.0	M	M	0.00	1.00	0.00
R3	1.0	L	L	0.00	0.00	1.00

**Table 5:** In case sensor gives wrong reading.

PM_2.5_ (µg/m^3^)		AQI		
**Sensor Data**	Predicted by CNN	Predicted by only BRB (only Sensor Data Are Considered)	predicted By Integrated Approach (BRB and CNN)	Actual Value
447	440.64	496.70	477.32	464
126 (wrong reading, accurate is 447)	440.64	208.34	263.96	
4	2.50	9.17	14.02	17
243 (wrong reading, accurate is 4)	2.50	259.48	159.53	

**Table 6:** Comparison of Reliability among three models.

Model	Sensitivity (%)	Specificity (%)	AUC
BRB based CNN	94.07	95.61	0.936
BRB	92.34	93.61	0.905
CNN	89.73	90.74	0.893
